# Supplementary material for: Digital Trends, Digital Literacy, and E-Health Engagement Predictors of Breast and Colorectal Cancer Survivors: A Population-Based Cross-Sectional Survey
Source: Int J Environ Res Public Health. 2023 Jan 13;20(2):1472. doi: 10.3390/ijerph20021472 (PMC9860554; doi:10.3390/ijerph20021472)
Supplement: Supplementary file 1 [file ijerph-20-01472-s001.zip › ijerph-2087867-supplementary.pdf]

Supplementary Materials: Patients' survey questionnaire

Digital trends, digital literacy, and e-health engagement predictors of breast and colorectal cancer survivors: a population-based cross-sectional survey.

Section (1) Socio-demographic Information

Question			
**1. Age /** years	☐ ☐		
**2. Gender:**	1. Male ☐		2 Female ☐
**3. Governance**	1. Amman	2. Irbid	3. Al Zarqa
	4. Al-Balqa	5. Al Mafraq	6. Karak
	7. Jerash	8. Madaba	9. Ajlun
	10. Aqaba	11. Ma'an	12. Tafila
**4. Marital status**			
**What is your marital status?**	Never married	1	
	Currently married	2	
	Separated	3	
	Divorced	4	
	Widowed	5	
	Refused	6	
**5. Employment**			
	Government employee	1	
	Non-government employee	2	

Which of the following best describes your **main work** status over the past 12 months?	Self-employed	3
	Non-paid	4
	Student	5
	Homemaker	6
	Retired	7
	Unemployed (able to work)	8
	Unemployed (unable to work)	9
	Refused	88

**6. Monthly income (JD's/month) ***		
**can you tell me what the average earnings Monthly Income (from all sources) of the household have been?**	less than 100 JD	1
	100-199 JD	2
	200-299 JD	3
	200-499 JD	4
	500 JD or more	5
	I don't know	6
	Refused to answer	7
**7. Education:**		
What is the **highest level of education** you have completed?	Illiterate (No formal schooling)	1
	Less than primary school	2
	Primary school completed	3
	Secondary school completed	4

	High school completed (Tawjihi)	5
	College/University completed	6
	Post graduate degree Masters/ PhD	7
	Refused	88
**8. chronic conditions?**		
**Do you suffer from any chronic conditions?**	**Diabetes**	**1.**
	**Hypertension**	**2.**
	**Other cardiovascular disease**	**3.**
	**Other, please specify (………)**	**4.**
	**I do not suffer from any chronic diseases**	**5.**

Section (2). Online information access and search

The following questions concern your use of internet to access and search for cancer supportive care information. "Cancer supportive care information" is used in its broadest sense and includes information about the disease, treatments and side effects, health professionals, prevention and well-being (healthy nutrition, exercise, stress management, etc.).

**1. To what extent are you able to seek for, access, interpret, and evaluate health information on the internet and use the knowledge obtained to solve health problems?"**	Very good	1
	Good	2
	Acceptable	3
	Poor	4
	Very poor	5
**2. Do you own a smartphone?**	1. Yes ☐	2. No ☐

	the answer is NO, please skip to section 3	
**3. Do you use mobile apps?**	1. Yes ☐	2. No ☐
**4. What types of apps do you use? (Check all that apply)**	Social media (e.g. Facebook, WhatsApp, YouTube, Instagram, Snapchat).	1
	Services (e.g. Google Maps, Uber, Food Delivery, e-fawteercom, Mobile Banking).	2
	Health Apps (e.g. Diet apps, Weight Management apps, Medication Reminders, Visita app).	3
**5. Are you open to receiving information about cancer management and communicate with your doctor using a mobile app similar to other services?**	Yes	1
	No	2
	Only if my doctor recommends it	3
	I am not sure	4

Section (3) : Cancer supportive care information

**1. Since you were diagnosed with cancer, have you used any online resources to know more about the disease?**	1. Yes ☐	2. No ☐
**2. Is there anyone around you that has**	1. Yes ☐	2. No ☐

**used the Internet to find health information for you?**		

**3. If YES, who (tick all that apply):**	Family members	1
	Friends	2
	Colleagues at work or neighbours	3
	Other, please specify (………………… …… .)	4
**4. When you search for health information, which sources do you use? (Check all that apply)**	My doctor	1
	Other health professionals (ex. Pharmacist, nurses)	2
	Internet	3
	Family/friends/Family/friends/another patients' advice	4
	Magazines/Journals/Books	5
	information leaflets	6
	Radio/Television	7
	Courses/Seminars	8
	Other, please specify	9
	Which resource was most useful? ………… …………………………………………………………	10

If you have not used the internet. Please answer question 5 and move to section 4

If you used the internet, please skip to question 6a

**5. For what reasons did you NOT use the internet to**	I did not have the need to look for information	1
	I did not know where/how to look on the Internet	2
	I do not trust the information on the Internet	3

**search information about cancer? Check all that apply.**	I find the information available on the internet confusing and/or difficult to Understand	4
	Do not know	5
**6.A If you used the internet to search for information about cancer, Which ONLINE sources do you usually get information from about cancer? (Check all that apply)**	Search engines (Google, Yahoo, Bing, etc.)	1
	Websites from official health organizations (e.g., king Hussein cancer centre)	2
	Online newspapers or magazines	3
	Wikipedia	4
	Patient online communities/networks.	5
	Social networks (e.g., Facebook, YouTube)	6
	Specific and dedicated apps for mobiles devices (tablets, phones).	7
	Patient organizations' websites	8
	Other, please specify (……………………)	9
**6.b Which resource was most useful? (………………………………………..)**		
**7.a <u>Since your time of diagnosis</u>, how often have you used the Internet to find cancer- related information?**	Less than 1-3 times a year	1
	Less than once a month	2
	2-3 times a month	3
	1-3 times a week	4
	Everyday	5
	Never	6
**7.b <u>Upon completion of treatment,</u> how often have you used the Internet to find cancer-related information?**	Less than 1-3 times a year	1
	Less than once a month	2
	2-3 times a month	3
	1-3 times a week	4
	Everyday	5
	Never	6

**8. Why have you looked for cancer-related information through the Internet? (Check all that apply)**	Because I was not satisfied with what doctor / health professionals told me	1
	Because I did not fully understand what doctor / health professionals told me	2
	Because I needed more information than what doctor / health professionals told me	3
	Because I did not want / I had no time to pay a visit to doctor / health professional	4
	Because I did not think doctor's advice was needed	5
	Because I wanted to check on the experience of people living my same health condition	6
	Because I wanted to share my experience with others	7
	Other	8
**9. What types of obstacles/barriers+ do you face when searching for cancer-related information? (Check all that apply)**	Difficulty to find/identify trustworthy sources of information "trustworthiness" refers to its perceived credibility of online sources whether regulated by medical institutions or not (e.g. patient forums, trusted blogs)	1
	Difficulty to assess the reliability of health information (e.g. information commercially oriented) Reliability is defined as whether health-related content is supported by medical evidence and/or approved by medical professionals or experts and referred to regulated websites with known affiliations.	2
	Difficulty to find information tailored to my specific needs	3
	Difficulty to cope with the quantity of information available	4
	The information lacks illustrations and visual information	5

	The information is difficult to understand/not clear enough	6
	The information is not detailed/specific enough	7
	The information is not available in a language I can speak	8
	I do not find what I am looking for	9
	Do not know	10
	Other, please specify (…………………………………………….)	11
	I did not face any obstacles	10
10. **What did you do to assess the reliability/trustworthiness of the cancer health information you found? (Check all that apply)**	Verify the information by asking my doctor or a health professional after performing the search.	
	verify results on other websites (e.g.)	
	Check other information sources (e.g. patient support groups, social media) to verify the results.	
	Ask the opinion of others (e.g., family, friends).	
	Do nothing.	
	Other, please specify (……………………………………………….)	

Section 4. Survivorship information openess

1. **At this stage, are you still interested in receiving information to help you in managing your condition?**	1. Yes ☐	2. No ☐
	Leaflet	1
	Verbally From HCP	2

<table>
<tr><td rowspan="3">2. In what format would you prefer to receive this information? (Check all that apply).</td><td>Via The Web</td><td>3</td></tr>
<tr><td>Using Mob Apps</td><td>4</td></tr>
<tr><td>Other, Give Options (……………………………………………)</td><td></td></tr>
<tr><td rowspan="2">3a. if a mobile app was designed containing the information you are interested in, would you use it?</td><td>1. Yes ☐</td><td>2. No ☐</td></tr>
<tr><td></td><td></td></tr>
<tr><td colspan="3">3b. if No, why not? ....</td></tr>
</table>

Thank you for your participation!
